# Supplementary material for: Modeling glioblastoma heterogeneity as a dynamic network of cell states
Source: Mol Syst Biol. 2021 Sep 16;17(9):e10105. doi: 10.15252/msb.202010105 (PMC8444284; doi:10.15252/msb.202010105)
Supplement: Supplementary file 6 — Source Data for Figure 5 [file MSB-17-e10105-s004.zip › Figure5A_sourcedata/GSEA_3017/hallmarks_stateE.GseaPreranked.1621934677158/gsea_report_for_na_pos_1621934677158.html]

Report for na\_pos 1621934677158 [GSEA]

| GS  follow link to MSigDB | GS DETAILS | SIZE | ES | NES | NOM p-val | FDR q-val | FWER p-val | RANK AT MAX | LEADING EDGE || 1 | HALLMARK\_EPITHELIAL\_MESENCHYMAL\_TRANSITION | Details ... | 35 | 0.64 | 3.78 | 0.000 | 0.000 | 0.000 | 265 | tags=91%, list=28%, signal=122% |
| 2 | HALLMARK\_UV\_RESPONSE\_DN | Details ... | 21 | 0.68 | 3.13 | 0.000 | 0.000 | 0.000 | 174 | tags=76%, list=18%, signal=91% |
| 3 | HALLMARK\_TNFA\_SIGNALING\_VIA\_NFKB | Details ... | 22 | 0.61 | 3.00 | 0.000 | 0.000 | 0.000 | 206 | tags=77%, list=22%, signal=96% |
| 4 | HALLMARK\_HYPOXIA | Details ... | 23 | 0.47 | 2.42 | 0.000 | 0.000 | 0.000 | 247 | tags=70%, list=26%, signal=92% |
| 5 | HALLMARK\_APOPTOSIS | Details ... | 25 | 0.36 | 1.88 | 0.000 | 0.010 | 0.019 | 206 | tags=60%, list=22%, signal=75% |
| 6 | HALLMARK\_IL2\_STAT5\_SIGNALING | Details ... | 16 | 0.42 | 1.81 | 0.011 | 0.013 | 0.029 | 261 | tags=69%, list=28%, signal=93% |
| 7 | HALLMARK\_P53\_PATHWAY | Details ... | 17 | 0.23 | 0.96 | 0.488 | 0.587 | 0.842 | 206 | tags=41%, list=22%, signal=52% |
| 8 | HALLMARK\_MTORC1\_SIGNALING | Details ... | 31 | 0.17 | 0.90 | 0.601 | 0.597 | 0.882 | 87 | tags=19%, list=9%, signal=21% |
Table: Gene sets enriched in phenotype **na **[plain text format]****
